# Management of Antenatal Agitation in a Tertiary Care Center in Central India: A Secondary Analysis

**DOI:** 10.7759/cureus.73968

**Published:** 2024-11-19

**Authors:** Priyash Jain, Varchasvi Mudgal, Rahul Mathur, Simran Sandhu, Virendra Pal

**Affiliations:** 1 Psychiatry, Mahatma Gandhi Memorial Medical College, Indore, IND

**Keywords:** agitation, antenatal, antipsychotics, management, psychopharmacology

## Abstract

Background

The presence of psychiatric symptoms in pregnancy is a common occurrence that requires swift and effective management to avoid harm to self, caregivers, staff, and, above all, the reliant fetus. However, there is a dearth of knowledge, practical guidelines, and research in the context of managing agitated states of antenatal patients. To bridge this critical knowledge gap, this research endeavors to illuminate the practices surrounding the management of agitated pregnant women with respect to psychiatric emergencies in a tertiary care hospital.

Aims and objectives

This study aims to study the pattern of psychotropic drugs used to manage agitation in antenatal patients in a tertiary care institute.

Methods

This retrospective record-based study was conducted in a tertiary care center in Central India to assess psychiatric referrals for the management of acute agitation in the department of obstetrics and gynecology. The consultation-liaison (CL) records of interdepartmental psychiatric referrals were assessed for a duration of one year between September 2022 and August 2023.

Results

The most frequently used treatment was intramuscular promethazine 25 mg, given to 32.20% of the participants. This was followed by intramuscular haloperidol 1.25 mg (14.69%) and tablet olanzapine 5 mg (10.17%). Tablet haloperidol 2.5 mg and intramuscular haloperidol 5 mg combined with promethazine 25 mg were each administered to 6.78% of the participants. Other treatments included tablet lorazepam 2 mg (6.21%), intramuscular lorazepam 2 mg (6.21%), intramuscular haloperidol 2.5 mg (9.60%), intravenous lorazepam 2 mg (2.26%), and tablet quetiapine 50 mg (1.13%). Verbal de-escalation was employed for 3.95% of the participants. None of the patients had to be restrained physically.

Conclusion

This study on antenatal agitation found promethazine followed by haloperidol as the most common treatment, used intramuscularly in low doses. Benzodiazepines like lorazepam were used sparingly, while verbal de-escalation proved effective in some cases.

## Introduction

The presence of psychiatric symptoms in pregnancy is a common occurrence that requires swift and effective management to avoid harm to self, caregivers, staff, and, above all, the reliant fetus [1]. The goal is to not only contain the agitation of the mother but also to minimize the risk of untoward events and pregnancy-related complications. The clinical characterization of significant agitation encompasses a spectrum of abnormal and excessive behaviors, such as verbal or physical aggression, purposeless motor activities, heightened arousal, and substantial disruption of the patient’s normal functioning [1]. Management of agitation has been well researched and boasts a significant evidence base; however, there is a dearth of knowledge, practical guidelines, and research in the context of managing agitated states of antenatal patients [2-5]. With the last update for practice guidelines being the consensus guidelines for the treatment of behavioral emergencies of 2001, there is a pressing need for the development of newer and evidence-based protocols for pregnancy [6].

This is highly relevant to primary care physicians, as the management of acute agitation in pregnant women is a scenario that may present in primary care settings. Primary care physicians are often the first to identify early psychiatric symptoms and must decide whether to initiate treatment, adjust care, or refer to specialists. Understanding the most common treatment strategies - such as the use of promethazine and haloperidol and the sparing use of lorazepam - helps them manage such cases safely, considering both maternal and fetal health. Furthermore, primary care physicians often serve as key coordinators between obstetrics, psychiatry, and other specialties, ensuring comprehensive and timely care for pregnant patients with psychiatric concerns.

This research work is an initiative to delve into the previously understudied emergency treatment in agitated pregnant patients. It is a fact, worth noting, that the safety, efficacy, and impact on fetal outcomes of utilizing polydrug use, like antipsychotic medications and/or benzodiazepines, in this special population remain largely unexplored. Notably, the revised Consensus Guidelines for Treatment of Behavioral Emergencies of 2005 make no mention of agitation management during pregnancy [2]. To bridge this critical knowledge gap, this research aims to assess the pattern of psychotropic drug use in the management of acute agitation in antenatal patients attended on a consultation-liaison basis by a psychiatrist in the department of obstetrics and gynecology.

## Materials and methods

This secondary analysis was conducted at a tertiary care institute in Central India to evaluate psychiatric referrals for the management of acute agitation in antenatal patients admitted to the department of obstetrics and gynecology after receiving the ethical committee approval on August 22, 2022 (letter no.: EC/MGM/Aug-22/51).

The consultation liaison (CL) records of interdepartmental psychiatric referrals were assessed for a duration of one year between September 2022 and August 2023. The department received 3406 psychiatric referrals during the study period, of which 373 referrals were from the department of obstetrics and gynecology for the management of acute agitation. These CL records were then screened further, and 184 referrals were excluded as they were for other indications like psychiatric disorders arising in post-natal and gynecological cases. This resulted in a sample of 189 referrals, of which a further 12 had to be excluded as the medical records either had inconsistencies, such as missing or contradictory information in the medical records, or incomplete data, leading to the exclusion of 12 cases from the sample of 189 referrals or incomplete data. A final sample of 177 referrals was obtained from which information was extrapolated.

A detailed assessment of the 177 CL records was conducted, and the data was recorded on separate sheets that comprised socio-demographic characteristics, including age, residence, educational status, religion, and marital status. The clinical data collected comprised gravida (Primi/multi), duration of current pregnancy, medical complications during pregnancy, psychiatric comorbidity as per ICD 10 classification, prior management before referral, and treatment used to manage agitation. The data was then analyzed using an open-source statistical analysis program, R 4.3.1.

## Results

Table 1 shows the mean age of the sample was 27.8 years. Out of 177 total participants, 57 (32.20%) were in the 24-29 years group, making the majority. It was observed that 64 (36.16%) out of the total received an education up to high school, followed by 58 participants (32.77%) up to middle school. Both the urban as well as rural areas had equal representation in the sample population, with 92 participants (51.98%) belonging to the urban region and 85 participants (48.02%) in the rural region. The majority of the participants were married, i.e., 174 (98.31%) of the total, and belonged to the Hindu religion, i.e., 97 (54.80%) of the total.

**Table 1:** Sociodemographic characteristics of the sample population

Sociodemographic variables		Frequency (n)	Percentage (%)	p-value
Age group				
(Mean = 27.8 years)	18-23 years	36	20.34	<0.05
	24-29 years	57	32.2	<0.05
	30-35 years	41	23.16	<0.05
	36-41 years	43	24.29	<0.05
Educational status	Illiterate	13	7.34	<0.05
	Primary school	34	19.21	<0.05
	Middle school	58	32.77	<0.05
	High school	64	36.16	<0.05
	Intermediate/diploma	7	3.95	<0.05
	Graduate	1	0.56	<0.05
	Professional degree	0	0	<0.05
Locality	Rural	85	48.02	<0.05
	Urban	92	51.98	<0.05
Religion	Hindu	97	54.8	<0.05
	Muslim	67	37.85	<0.05
	Sikh	3	1.69	<0.05
	Others	10	5.65	<0.05
Marital status	Married	174	98.31	<0.05
	Unmarried	2	1.13	<0.05
	Divorced/separated	1	0.56	<0.05

Table 2 summarizes the clinical characteristics of the sample population. The majority were multigravida (75.7%) and in the third trimester of pregnancy (83.05%). Most participants had no medical complications (57%), though hypertension (22%) and eclampsia (19%) were the most common issues. Psychiatric diagnoses were primarily delirium not induced by alcohol or psychoactive substances (53.11%), followed by unspecified nonorganic psychosis (19.21%) and acute transient psychotic disorders (7.34%). A small percentage had persistent delusional disorders (2.26%) or no psychiatric diagnosis (3.95%).

**Table 2:** Clinical characteristics of the sample population

Clinical characteristics	Frequency (n)	Percentage (%)	
Gravida	Primigravida	43	24.3
	Multigravida	134	75.7
Current duration of pregnancy	First trimester (0-12 weeks)	7	3.95
	Second trimester (13-24 weeks)	23	12.99
	Third trimester (>28 weeks)	147	83.05
Medical complications during pregnancy	Nil	101	57
	Eclampsia	34	19
	Hypertension	39	22
	Hypothyroidism	13	7.3
	Diabetes mellitus	21	12
Psychiatric diagnosis (as per ICD 10)	Delirium, not induced by alcohol and other psychoactive substances (F05)	94	53.11
	Acute and transient psychotic disorders (F23)	13	7.34
	Severe depressive episode with psychotic symptoms (F32.3)	8	4.52
	Unspecified nonorganic psychosis (F29)	34	19.21
	Other mental disorders due to brain damage and dysfunction due to physical disease (F06)	17	9.6
	Persistent delusional disorders (F22)	4	2.26
	Nil	7	3.95

Table 3 details the various treatments administered to manage agitation in the sample population. The most frequently used treatment was intramuscular promethazine 25 mg, given to 57 participants (32.20%). This was followed by intramuscular haloperidol 1.25 mg given to 26 participants (14.69%) and tablet olanzapine 5 mg given to 18 participants (10.17%). Tablet haloperidol 2.5 mg and intramuscular haloperidol 5 mg combined with promethazine 25 mg were each administered to 12 (6.78%) of the participants. Other treatments included tablet lorazepam 2 mg in 11 (6.21%), intramuscular lorazepam 2 mg in 11 (6.21%), intramuscular haloperidol 2.5 mg in 17 (9.60%), intravenous lorazepam 2 mg in 4 (2.26%), and tablet quetiapine 50 mg in 2 (1.13%) participants. Verbal de-escalation was employed for seven (3.95%) of the participants. None of the patients had to be restrained physically.

**Table 3:** Treatment administered for the management of acute agitation

Treatment administered	Frequency (N)	Percentage (%)	p-value
Non-pharmacological	7	3.95	<0.05
Verbal de-escalation	7	3.95	<0.05
Oral route	43	24.29	<0.05
Tablet haloperidol 2.5 mg	12	6.78	<0.05
Tablet olanzapine 5 mg	18	10.17	<0.05
Tablet lorazepam 2 mg	11	6.21	<0.05
Tablet quetiapine 50 mg	2	1.13	<0.05
Intramuscular route	123	69.49	<0.05
Intramuscular promethazine 25 mg	57	32.2	<0.05
Intramuscular haloperidol 1.25 mg	26	14.69	<0.05
Intramuscular haloperidol 2.5 mg	17	9.6	<0.05
Intramuscular haloperidol 5 mg + promethazine 25 mg	12	6.78	<0.05
Intramuscular lorazepam 2 mg	11	6.21	<0.05
Intravenous route	4	2.26	<0.05
Intravenous lorazepam 2 mg	4	2.26	<0.05

## Discussion

The current study was conducted in a tertiary care institute with the aim of assessing the pattern of management techniques being used in cases of antenatal agitation. The study reveals that in cases of antenatal agitation, the most commonly used treatment modality was intramuscular promethazine, followed by intramuscular haloperidol in doses ranging from 1.25 mg to 2.5 mg. Benzodiazepine had been used sparingly, and in cases where the use of benzodiazepine was warranted, lorazepam was the preferred agent, which was used either orally or intramuscularly. No other benzodiazepine was used. There were four cases where intravenous lorazepam was necessitated. Seven patients were managed by verbal de-escalation while none of the patients had to be physically restrained.

Intramuscular injections were the predominant mode of treatment, likely due to their rapid onset of action, which is critical in acute agitation. Promethazine was the most administered drug, often chosen for its sedative properties and adjunctive use with antipsychotics like haloperidol [7]. The combination of haloperidol and promethazine highlights a synergistic approach to managing both psychotic and agitation symptoms.

Oral medications are preferred for their ease of administration and the patient’s ability to self-administer, promoting autonomy and comfort. Olanzapine was the most frequently used oral antipsychotic, likely due to its efficacy and relatively favorable safety profile during pregnancy [8]. Lorazepam, an anxiolytic, was also commonly used, reflecting the need to manage anxiety components of agitation. A few other psychotropics like risperidone, chlorpromazine, aripiprazole, etc. were lacking in the pattern of usage. This abstinence is attributed to availability in the institution, lack of injectable preparation, and absence of sedative properties, which are often required in the management of agitation. Non-pharmacological interventions, including verbal de-escalation and other supportive measures, were used in seven patients each. These approaches are crucial first-line strategies due to their minimal risk to the fetus. However, their limited efficacy in severe cases of agitation necessitates the use of pharmacological interventions.

The literature on the management of antenatal agitation is scarce. One study by Ladavac et al. assessed the pattern of management of agitated pregnant women and found that haloperidol was the most frequently administered psychotropic medication, while risperidone was the second most commonly administered medication. Benzodiazepine was used alone or in combination with haloperidol whenever required [1]. In our study, we observed that injectable promethazine followed by haloperidol and benzodiazepine was the most common medication for the management of antenatal agitation.

Management of antenatal agitation poses a significant challenge as it requires careful consideration of the safety of the mother as well as the unborn and dependent child [9]. Extensive guidelines for the management of agitated patients in emergency settings are available; however, there is a dearth of research into the management of agitation in antenatal patients [2,10,11].

Pregnancy results in alterations in physiological and pharmacokinetic parameters resulting from altered glomerular filtration rates and expansion of plasma volume. These changes, in turn, affect the distribution, absorption, metabolism, and excretion of drugs [12,13]. This necessitates the use of medications with shorter half-lives so that they may not accumulate in maternal and fetal tissues [14].

The use of medications during pregnancy poses risks to the fetus, which can vary depending on the timing and type of drug used. Teratogenic drugs taken during the first trimester can cause malformations, while those used later in pregnancy can lead to complications during delivery and adverse outcomes for the neonate. For instance, benzodiazepines, when used late in pregnancy, have been linked to increased rates of spontaneous intrauterine fetal demise, higher incidences of cesarean delivery, and a greater likelihood of neonatal admissions [15,16]. Additionally, the risk of a relapse in the mother's mental disorder due to inadequate treatment further complicates the situation.

The Maudsley Prescribing Guidelines underscore the lack of research evidence on rapid tranquilization in pregnancy and then posit the use of short-acting benzodiazepine or promethazine, although with a risk of adverse outcomes to the fetus [7]. The British Association for Psychopharmacology (BAP) guidelines conclude on similar lines that the evidence regarding the risks and benefits of psychotropic medication in the perinatal period has many weaknesses, particularly the multiple confounding factors. This makes definitive statements about risks and benefits impossible in most cases. It is therefore usually necessary to generalize evidence of efficacy from studies in non-perinatal populations.

Also, in most emergency situations, the benefits of preventing the patient from harming herself and others due to agitation far exceed the risks of exposure to the medication. It has also been suggested that a single dose of an antipsychotic and/or benzodiazepine is generally considered low-risk [17]. In our study, all the patients, barring four, received only a one-time dose of the prescribed psychotropic. Physical restraint during pregnancy also poses a significant risk to the agitated antenatal patient. Women in their advanced pregnancy may develop obstruction of venous return to the heart and supine hypertension syndrome if placed in the supine position for prolonged duration [1]. Placing women in a prone position poses a risk to the fetus. Hence, physical restraint must be reserved only as a last resort and for the minimum duration possible. Also, if possible, the patient should be placed in a reclined position [14].

Given the frequent occurrence of psychiatric distress in pregnant women, primary care providers are often the first point of contact for these patients. Early recognition of signs of agitation and timely intervention can significantly mitigate risks to both the mother and the fetus, emphasizing the importance of knowledge about psychiatric conditions and their management in pregnancy.

The study has several limitations. As a secondary analysis, it relied on previously recorded data, a small part of which had to be rejected due to incomplete or inconsistent data, potentially impacting the accuracy of the findings. The study was conducted at a single tertiary care institute, which limits the generalizability of the results. Additionally, there was no follow-up data to assess long-term treatment outcomes. The severity of agitation was also not recorded as the cases were seen as referrals in the department of obstetrics and gynecology. Many psychotropics were lacking in the pattern of usage due to various factors. Thus, all of the above limitations impact the generalizability of the findings. To address the gaps identified in managing antenatal agitation, future research should focus on developing updated, evidence-based clinical guidelines tailored to pregnant patients. Longitudinal studies are needed to evaluate the long-term outcomes of current treatment approaches, including both pharmacological and non-pharmacological interventions. Implementing standardized protocols and training for healthcare professionals will improve the management of antenatal agitation.

## Conclusions

This study on antenatal agitation found promethazine followed by haloperidol as the most common treatment, used intramuscularly in low doses. Benzodiazepines like lorazepam were used sparingly, while verbal de-escalation proved effective in some cases. The use of physical restraint was minimal. The management of antenatal agitation involves a multi-faceted approach, with a strong reliance on intramuscular psychotropics for immediate control. While pharmacological treatments are indispensable for severe cases, the integration and enhancement of non-pharmacological approaches remain crucial.

Future research should focus on optimizing treatment protocols to balance efficacy and safety for both the mother and fetus. This study revealed important lacunae in the management of standardized guidelines for females with antenatal agitation. We found significant variations in the pattern of psychotropic drug use amongst antenatal patients, underscoring the pressing need for standardized guidelines. The lack of up-to-date protocols in treatment may impact both maternal and fetal outcomes. There is a rising trend of psychotropic use, primarily antipsychotics, for the management of agitation, which demands the need to develop and implement evidence-based guidelines for the management of antenatal aggression. This would ensure that healthcare providers could make uniform decisions, ultimately improving the quality of care for antenatal patients.
